# State Medical Association Matters

**Published:** 1905-01

**Authors:** 


### State Medical Association Matters.

### THE BILL FOR A STATE BOARD OF HEALTH.

The following is the bill agreed upon by the Committee on Public Policy and Legislation in conference with representatives from numerous county medical societies in Austin November 14th and 15, 1904. It is published for the first time, and reprints have been sent to the Secretary of every county medical society in the State. The Attorney General was requested by State Health Officer Tabor to pass upon the bill, but General Bell declined to do so. He wrote Dr. Tabor as follows:

"I note that you say that the measure is to be introduced in the Thirtieth Legislature. Long before that time I will have ceased to be Attorney General of the State, and I do not care to express any opinion with reference to a matter that could not possibly come before me in my official capacity."

The opinion of the Attorney General elect, Judge Davidson, will be asked upon the bill. It will then be reported to the House of Delegates at the next meeting of the State Medical Association for final action and instructions:

### AN ACT

To carry into effect Section 32 of Article XVI of the Constitution of the State of Texas in relation to a State Board of Health and Vital Statistics; and to better protect the public health to create and establish a State Board of Health; to define its duties and fix the compensation of its members and employes; to create county and municipal health officers and define their duties; to authorize the State Board of Health to prepare, promulgate and enforce, under suitable penalties for violation of its provisions, a sanitary code for the State of Texas covering every phase of the subject, including quarantine; to provide for general sanitary supervision of counties and municipalities and to repeal all laws and parts of laws in conflict with this act; and declaring an emergency.

SECTION 1. *Be it enacted by the Legislature of the State of Texas:* That a State Board of Health and Vital Statistics is hereby created and established, to consist of three (3) legally qualified physicians of good standing, graduates of regularly chartered and legally constituted medical colleges, the State Health Officer appointed under existing laws, being ex-officio a member and President of the Board and its executive officer, the other two members of the Board to be physicians appointed by the Governor from a list of six names to be submitted to him by the State Medical Association of Texas.

SEC. 2. The Governor shall, by and with the consent of the Senate, on or before the 10th day of May, 1907, appoint the members of the Board as heretofore provided, the President of the Board to receive an annual salary of $3600, the other two members to receive no salary, but each to be allowed $10 per day for each and every day they are in attendance upon meetings of the Board, including the time spent in transit, and 5 cents per mile going to and returning from such meetings, to be paid on their voucher, approved by the Governor and the President of the Board, by warrant drawn on the Comptroller against the general appropriation provided for this purpose.

SEC. 3. A majority of the members of the Board shall constitute a quorum for the transaction of business. The Board shall meet at Austin on the first Tuesday after appointment and commission, and every three months thereafter on a day to be fixed by the Board, or whenever such meeting is necessary, timely notice of such meetings having been given every member of the Board. They shall meet on the call of the President or on demand of two members.

The office of the Board shall be in the Capitol at Austin, and the Board shall be furnished with all necessary equipment, including laboratory supplies, books, stationery, blanks, etc., as other departments of the State Government are furnished.

SEC. 4. The Board shall, at its first meeting after commission, appoint assistants as follows: (1) A Registrar of Vital Statistics, to receive a salary of $2000 per annum. It shall be his duty to collect, record, tabulate and publish in the yearly report of the Board hereinafter provided for, the vital and mortuary statistics of the State, thus making effective the law on the subject passed by the Legislature of 1903, and shall, from time to time, as directed by the Board, prepare, publish and disseminate among the people for their information and protection, such literature as may enlighten the public upon sanitary matters and the causes and means of prevention of diseases. (2) A Chemist and Bacteriologist, who shall be learned in chemistry, pathology and bacteriology. He shall receive a salary of $2000 per annum. He shall give as much of his time as is necessary to the duties of his position, make all examinations and analyses of matters referred or submitted to him by the Board, and shall report the result of such examinations in writing to the Board, which reports shall be incorporated in the annual report of the Board hereinafter provided for. (3) A Secretary to the President, who shall receive a salary of $1200 per annum. (4) Two clerks, who shall each receive a salary of $900 per annum. (5) An Inspector, who shall receive $1800 per annum, and (6) a porter, who shall receive $360 per annum.

The sum of ——— dollars, or as much thereof as is necessary, is hereby appropriated and set aside out of the general revenue of the State to pay the salaries and expenses provided for in this bill.

SEC. 5. The members of the Board of Health shall hold their offices for a term of two years and until their successors are appointed, unless sooner removed for cause. They shall qualify and be commissioned in the manner provided by the laws of Texas for all other officers of the State.

SEC. 6. The State Board of Health shall have the general supervision of the interests of the health and lives of the citizens of the State. It shall make a study of the causes of epidemic diseases within the State and have the direction and control of all quarantine to prevent the entrance of contagious and infectious diseases from without, and the direction and control over all sanitary and quarantine measures for dealing with infectious and communicable diseases within the State in order to suppress and prevent the spread of the same.

SEC. 7. The President of the Board shall have charge of and superintend the administration of all maritime, international and internal quarantine and disinfection for the protection of the State from contagious and infectious diseases according to the regulations established and promulgated by the Board. He shall have power at all times to issue all orders and warrants and to take all necessary steps to execute the sanitary laws of the State and the rules, ordinances and regulations of the Board made under authority of this act. He shall perform certain other duties hereinafter stipulated.

The State Board is authorized to employ special inspectors and assistants to supervise or assist in sanitary inspection or investigation when necessary, but no more than $2000 shall be expended for this purpose in any one year.

SEC. 8. The Board shall prepare, or cause to be prepared, a sanitary code for the State of Texas, which shall contain and provide rules and regulations and ordinances of a general and special nature for the improvement and amelioration of the hygienic and sanitary condition of the State. On adoption of said code by the Board it shall be published at length and in full in at least two newspapers in the State, on ten consecutive days, and there shall also be printed and published in pamphlet form such number of copies as may be necessary for distribution for the information of health boards, health and sanitary officers, and the public generally. Said code shall cover and provide for, especially, land and maritime quarantine regulations; the reporting, care and management of infectious, contagious and communicable diseases; it shall regulate the manner of keeping and reporting and tabulating vital and mortuary statistics; it shall provide for affording facilities for vaccination, provided the same shall not be made compulsory except in cases of children attending public schools; it shall regulate the carriage and transportation of persons, freight and dead bodies brought into the State or transported through or into the State so far as the same may affect the public health; it shall provide for the carrying out of the laws of the State in regard to the adulteration of articles intended for human food or consumption; it shall provide for the inspection of meats, milk, coal oil, and other articles affecting the public health and safety where and when the same may be brought from one county to another, or from outside of the State, leaving to local boards hereinafter provided, the regulation of the sale or offering for sale of said articles within the county or municipality to which the same may be brought, and said code shall contain general rules in regard to such health, sanitary and hygienic subjects, as can not in the opinion of the State Board of Health, be efficiently and effectively regulated by the local boards. It shall devise and execute, under suitable penalties for violation thereof, ordinances to prevent the pollution of all water streams and supplies. It shall make and have power to enforce, under penalties fixed by the Board, ordinances to regulate sanitary policing of premises, public or private, jails, hospitals, public buildings, shops, factories, school houses, slaughter houses and yards, for the removal of known and possible causes of disease; to abate nuisances; to cause disinfection of all infected houses, public or private, and of sleeping cars and other railroad cars, and of all public conveyances, these means of prevention to be taken by the owners or agents of said premises or conveyances at their own cost. It shall regulate the sale of secret or patent medicines, prohibiting the sale of such as contain alcohol or opium or cocaine or other substances deleterious to the public health.

SEC. 9. The Board shall have power to appoint inspectors when necessary and fix the salaries thereof. All inspectors and officers of the said Board shall have power to arrest, without warrant, all persons violating the provision of any rule or regulation of the said Board, when such violation has occurred or is occurring within the sight, view or personal knowledge of such inspector or officer; and in all cases where such violation may not have occurred within the sight, view or personal knowledge of said inspector or officer, said functionary shall have only the right to arrest in execution of a warrant duly issued by the President of the Board.

It is hereby made the duty of all sheriffs, their deputies, constables and their deputies, police officers of towns and cities, and all other peace officers, to assist in the arrest or apprehension of all persons violating the articles of any rule or regulation of the State Board of Health; to themselves arrest and apprehend all offenders committing the offense in their view or sight, or within their personal knowledge.

SEC. 10. The members of the State Board of Health and every person duly authorized by them may, without fear or hindrance, enter, examine and inspect all grounds, erections, vehicles, structures, public buildings and places.

SEC. 11. Be it further enacted that the County Commissioners of each and every county in the State shall, immediately after the promulgation of this act, appoint a resident, duly licensed physician of the county, of recognized character and standing, to be County Health Officer, and in default of making such appointment within thirty days after notice to do so by the State Board of Health, the State Board of Health shall appoint a County Health Officer from among the legalized practitioners of medicine of the county. The County Health Officer shall serve two years from the date of his appointment, and shall be paid by the county such salary as may be agreed on.

Said County Health Officer shall have sanitary supervision of all the territory embraced in his county outside the limits of incorporated towns and cities, which shall be under the jurisdiction of their respective municipal health officers hereinafter provided for. The County Health Officer shall exercise the powers and perform the duties prescribed for him by the Commisioners Court, and shall also enforce the sanitary and quarantine rules and regulations prescribed by the State Board of Health for the protection of the public health, the prevention and suppression of diseases, and shall be under the direction of and subject to the State Board of Health, and shall obey its instructions, in default of which he shall be fined not to exceed $100, at the discretion of the State Board of Health for neglect or failure to so do, and the County Health Officer may be removed by said State Board for continued or repeated neglect of duty or incompetency. In event of such removal the State Board of Health shall appoint the County Health Officer from among the legal practitioners of medicine in the county.

SEC. 12. Be it further enacted, etc., that the council or legislative body of each and every incorporated town or city in the State shall appoint a City Health Officer, or establish and organize a town or city board of health, in accordance with their own ordinances, the chairman of which board shall be City Health Officer. In default of making such appointment within thirty days after notice to do so by the State Board of Health, the said Board shall appoint a City Health Officer from among the legalized practitioners of medicine of the municipality. The City Health Officer shall have sanitary supervision of the territory embraced in his municipality. He shall exercise the powers and perform the duties prescribed for him by the legislative body of his town or city, and shall also enforce the sanitary and quarantine rules and regulations prescribed by the State Board of Health for the protection of public health, the prevention and suppression of diseases, and shall be under the direction of and subject to the State Board of Health, and shall obey its instructions, in default of which he shall be fined not to exceed $100, at the discretion of the State Board of Health for neglect or failure to do so, and the City Health Officer may be removed by said State Board for continued or repeated neglect of duty or incompetency. In every such removal the State Board of Health shall appoint a City Health Officer from among the legal practitioners of medicine of the municipality.

SEC. 13. Be it further enacted, etc., that said county and health officers shall have power and authority to pass health and sanitary ordinances for defining and abating nuisances dangerous to the public health; to regulate drainage and ventilation, with reference to human habitations and places of business and public resort; to regulate the carrying on of trade and business injurious to public health; for the disposition of dead animals, fecal matter and garbage; to regulate the erection of buildings, with due regard to the filling of lots and the grading thereof, and the arrangement of said building; for the vacation of, disinfection of, or demolition of buildings, when necessary for the protection of public health; for the registration of births, deaths, and marriages, and the keeping of vital statistics, to be registered and reported to the State Board of Health, under its instructions and regulations, and generally all health and sanitary ordinances necessary and incident to proper local sanitation of the county, city or town in which they exercise their powers.

They shall act under the supervision and advice of the State Board of Health, and shall pass no ordinance in conflict or inconsistent with the powers and duties of the State Board of Health, and shall furnish information such as the State Board of Health may require, the same to be embodied in the annual report of the State Board.

SEC. 14. All necessary expenses, costs and charges of local sanitation shall be borne by the county, city or town in which the local boards shall be established, and in case the fiscal authorities shall refuse to budget for, appropriate or pay the same, the State Board shall have the right to the writ of mandamus before a court of competent jurisdiction to compel the proper action of said county, city or town authority.

SEC. 15. Be it further enacted, etc., that County and City Health Officers shall follow the rules and regulations promulgated by the State Board of Health in the management of local epidemics of infectious and contagious diseases, and in the event that such diseases exist in any locality, which in the opinion of the President of the State Board of Health threatens to invade other localities, the President of the State Board of Health shall declare the fact and instruct the proper county, city or town health officers as to measures of isolation, quarantine intercourse, etc., to pursue calculated to prevent the spread of the contagion or infection. In the event said local authorities shall fail or neglect to comply with the orders of the State Board of Health within a reasonable time, or in so acting shall fail to do in a manner satisfactory to the State Board, said Board, through its President, shall take charge of the case and manage the same through its own officers and employes. All expenses, costs and charges incurred in such management, control and supervision shall be borne and paid by the county or municipality in which the case or cases may be, and the failure of the county or municipality to reimburse the State Board for the amount of its expenditures incurred in such cases, the State Board of Health shall have the right to proceed by writ of mandamus in any court of competent jurisdiction to compel the payment of the same.

SEC. 16. Be it further enacted, etc., that all laws and parts of laws in conflict with, inconsistent with, or superceded by the provisions of this act are hereby repealed, but all laws or parts of laws, or city ordinances, State Board of Health ordinances, State and municipal rules and regulations now existing and not in conflict with, or inconsistent with or superceded by the provisions of this act are continued in full force and effect, and this act shall not be construed or interpreted so as to deprive the State Health Officer, or the local boards of health of any powers or authority, except in so far as these powers and authorities may be modified or changed by the provisions of this act, and then only to the extent of the modification or change.

SEC. 17. The Governor may remove any member of the State Board of Health for cause.

SEC. 18. The State Board of Health shall make and publish a full report of its work on the 1st of September of each year, as all other reports are made to the Governor and the Legislature.

SEC. 19. The fact that there is no uniform and efficient law for the suppression of disease other than those of foreign origin, and no effective system of preserving and utilizing the vital statistics of the State, creates an emergency and an imperative public necessity that the constitutional rule requiring bills to be read on three several days be suspended, and that this act take effect and be in force from and after its passage, and it is so enacted.

